# A first city-wide early defibrillation project in a German city: 5-year results of the Bochum against sudden cardiac arrest study

**DOI:** 10.1186/1757-7241-18-31

**Published:** 2010-06-15

**Authors:** Christoph Hanefeld

**Affiliations:** 1 Emergency Medical System of the city of Bochum, Brandwacht 1, 44894 Bochum, Germany

## Abstract

**Background:**

Immediate defibrillation is the decisive determinant of prognosis in patients suffering from cardiac/circulatory arrest caused by ventricular fibrillation (VF). Therefore, various national and international associations recommend that first responders use defibrillators as soon as possible and also recommend public access to early defibrillation programmes. Here we report the results of the first city-wide early defibrillation project in a large German urban area.

**Methods:**

There were 155 automated external defibrillators (AEDs) put into operation in the Bochum municipal area, and 6,294 people took part in cardiopulmonary resuscitation (CPR) and AED training. Free, accessible AEDs were installed in places with large volumes of people. Additionally, emergency forces were progressively equipped with AEDs.

**Results:**

Twelve AED administrations prior to the arrival of an emergency physician were recorded and analysed over a period of 5 years (08/2004-08/2009). Rhythm analysis via AED demonstrated VF in seven cases, non-malignant dysrhythmias in four cases and asystole in one case. Two of the seven patients with VF were successfully defibrillated and survived cardiac/circulatory arrest without any neurological sequelae. Eight of the 12 AED applications were performed by laymen. The mean time between switching the unit on and applying the electrodes to the patient was 39 seconds (SD +/-20 sec). On average, another 20 seconds elapsed before the AED recommendation of "shock delivery" was displayed, and a total of 96 seconds elapsed before shock administration (± 56 sec).

**Conclusion:**

Consistent with other reports, our findings show that the organisation of a city-wide initiative by a project office combining public access and first-responder defibrillation programmes can be safe, feasible and successful. Our experiences confirm that strategic planning of AED placement is a prerequisite for successful, cost-effective resuscitation.

## Introduction

Cardiovascular disease is the most common cause of death in individuals over the age of 40 years [1]. In the US, approximately 250,000 individuals die from cardiac/circulatory arrest annually, and the most common dysrhythmia is ventricular fibrillation VF [2]. In Europe, the overall incidence for all-rhythm arrests is estimated as 37.72 per 100,000 person-years [3]. Notably, less than 5% of patients survive an out-of hospital cardiac/circulatory arrest [4]. Different approaches have been pursued in the past to train the population in recognising cardiac/circulatory arrest and applying basic CPR measures; attempts have also been made to improve emergency medical services (EMS) care [5].

The decisive determinant of prognosis in patients suffering from cardiac/circulatory arrest caused by VF is immediate defibrillation. The chance of survival in these patients depends directly on the time elapsed between cardiac/circulatory arrest and defibrillation [6-8]. Therefore, different national and international associations recommend that first responders use defibrillators as soon as possible, and they recommend public access to early defibrillation programmes [9,10]. If CPR is not performed, the chance of survival decreases by 7-10% each minute [11]. In the European population, approximately, 275,000 persons would experience all-rhythm cardiac arrest treated by the EMS with 29,000 persons surviving to hospital discharge [3]. With this in mind, public health programmes have been initiated in different areas in the past 10 years, focusing on CPR initiatives based on the implementation of early defibrillation in different institutions, public buildings and recreational facilities.

Automated external defibrillators (AEDs) developed for this purpose can be operated by medical laymen. These devices possess an algorithm that independently recognises a malignant dysrhythmia requiring shock delivery and that enables shock administration" [12]. In pilot projects (e.g., airports, casinos, wide-bodied aircraft and local programmes supported by the police), survival rates of 49-74% have been achieved following the implementation of such early defibrillation programmes [13-15]. These results have led to the initiation of further, mainly local, programmes in highly frequented public places. On an international level, emergency medical services (EMS) differ dramatically in terms of control-room organisation, shared responsibilities, allocation of tasks and distribution of resources. Thus, the organisational structures of existing regional defibrillator programmes, most of which are pilot projects, differ considerably. Therefore, supranational comparative data of procedures, survival rates and survival type are available to only a limited extent. Two different concepts of early defibrillation are practiced: 1) "public access defibrillation programmes," which aim at facilitating defibrillation among the general public; and 2) "first responder defibrillation programmes," which provide training and equipment for professionals to defibrillate the cardiac arrest victims they encounter during their work. In public access defibrillation programmes, AEDs are installed in places with high volumes of people. In case of an emergency, individuals passing by (e.g., in airports and central stations) are able to use the provided AEDs in a timely manner without receiving thorough training. In first-responder defibrillation programmes, AEDs are used by trained first responders (e.g., security staff, police officers and accompanying personnel in aircraft and trains) who are immediately involved in a circulatory arrest as an eyewitness or are the first to arrive. Both concepts are currently approved by the German Medical Association (Bundesärtzekammer). Basic areas under investigation include integration of the local EMS and medical quality management [16].

In this study, we report results gathered in an early defibrillation project in the central Ruhr area over a period of five years. The project, called "Bochum against Sudden Cardiac Arrest," reflects an authentic situation of a system mainly initiated and supported by public institutions and volunteers and implemented with the support of the local EMS.

In addition to the main outcome measures "number of successful defibrillations" and "survival", we report the frequency of AED use and the timeline of AED application. We also report experiences with the implementation of the programme in Bochum, including the preceding training and an estimation of the costs of the project in relation to the number of lives saved.

## Methods

Bochum is a city of 380,000 inhabitants in the central Ruhr area. As a city-wide programme, the "Bochum against Sudden Cardiac Arrest" initiative was implemented in 2003. The initiative is funded by the city of Bochum, the local EMS, various health insurance agencies and medical representatives from hospitals and in private practice. It is also supported by numerous public and private institutions. The concept was designed to facilitate dynamic improvement and enable the incorporation of findings emerging in the course of the project. It involves a combination of the principles of "public access" and "first-responder defibrillation programmes". Accessible AEDs were successively installed in areas with high volumes of people (e.g., public buildings, businesses and event centres) in the municipal area from 2003 onwards. Additionally, emergency forces and medically educated staff (e.g., fire brigades and private practices) were progressively equipped with AEDs. Individuals without a medical background who worked near the publicly accessible AEDs, in addition to emergency forces, were instructed in the use of the AEDs and were familiarised with the basics of CPR by means of training seminars. A structured, one-hour AED training session was offered to small groups (maximum of eight people). Such sessions included basic knowledge of cardiovascular event recognition and CPR, and participation was certified and numerically recorded. The training contents and local conditions were calibrated with other training seminars from, for example, the German Red Cross.

The project is headed by the medical director of the EMS, and the EMS is responsible for project coordination and quality management. Project-related inquiries are addressed via a central hotline. The AEDs were purchased by individual institutions, societies and private practices with their own financial means. This had an impact on the distribution pattern, as the allocation of the AEDs depended on the involved institutions rather than strategic planning. Based on targeted contacting of particular institutions and businesses, however, it was possible to install AEDs in places considered to be high-risk (e.g., event centres and shopping centres).

The project office is informed about the use of an AED in the municipal area via a hotline or the EMS control room. Timely recording and medical evaluation of events are performed by the project management in the scope of quality management.

Since April 2009, the nearest AED available to the site of an event can be seen on a screen in the control room. Furthermore, the telephone numbers of the trained first responders are displayed, and they can be immediately called in case of an emergency.

The reported data are a descriptive report of the implementation and clinical outcomes of an early defibrillation programme in a German urban area over five years.

## Results

**Figure 1:**
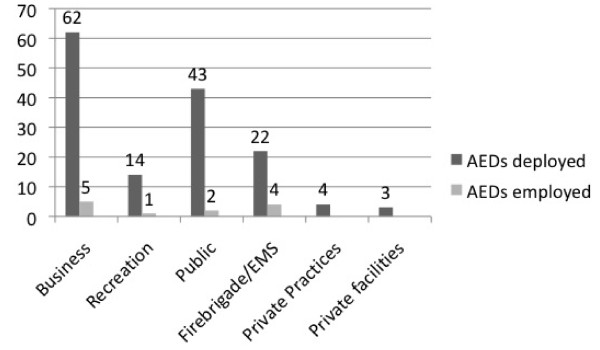
**AED Sites and Users, Total Number of AEDs Placed = 155**.

Following the initiation of the programme at the end of 2003, 155 AEDs were successively put into operation in the Bochum municipal area up to the present day (for the distribution, see Fig. 1), and 6,294 first responders were trained.

In total, 12 AED administrations after circulatory collapse were recorded and analysed during the period of data collection (08/2004 - 08/2009) **(see Fig. **1).

Ten AED administrations were preceded by the observed collapse of the subjects, whereas two administrations were preceded by an unobserved collapse. In all 12 cases, the AEDs were used prior to the arrival of the emergency physician who was called to ensure further medical treatment of the patient.

There were seven cases of VF, four cases of non-malignant dysrhythmia and one case of asystole (**see Table **1). The patients who suffered non-malignant heart rhythms were responsive at the time of EMS arrival.

**Table 1:** Clinical outcome depending on underlying rhythm disturbance and type of resuscitation

Initial rhythm		Last rhythm documented by AED	CPR performed	Clinical course	Time (sec.) Switch-on to shock delivery
**VF**	VF	sVT	3 cycles	discharged from hospital	93
	VF	SR	-	discharged from hospital	59
	VF	VF (low-amplitude)	1 cycle	death in hospital	190
	VF	VF	2 cycle	death in hospital	83
	VF (low-amplitude)	asystole	-	exitus letalis	49
	VF (low-amplitude)	asystole	-	exitus letalis	39
	VF	asystole	1 cycle	exitus letalis	149
**non-malignant dysrhythmia**	SR (bradycardia)	SR	-	discharged from hospital	No shock recommended
	SR	SR	1 cycle	discharged from hospital	No shock recommended
	AV junctional escape rhythm	idem	-	discharged from hospital	No shock recommended
	AV junctional escape rhythm	idem	-	discharged from hospital	No shock recommended
**asystole**	asystole	asystole	3 cycle	exitus letalis	No shock recommended

The two patients with VF who could be discharged from the hospital survived without any neurological damage (**see Table **2). In both cases, an AED was directly available at the emergency site (< 100 metres); it was used by laymen onsite, and AED shock delivery led to successful conversion into a palpable rhythm. In the case of the other five patients with VF, an AED was not directly available at the emergency site but was retrieved by a first responder (in one case, the first responder was a trained passerby, and in four cases, the AED was used by alerted firemen). Thus, the AED could only be used after a time delay of 4-6 minutes. In these cases, no conversion into a palpable rhythm could be achieved. In two cases, the shock was administered after 149 and 190 seconds, and the automated speech announcement was ignored, despite a recommendation for shock delivery. It is presumable that in these cases, the users were reluctant to deliver the shock. In the four cases in which anamnestic data about the circulatory collapse were available and non-malignant dysrhythmia could be demonstrated, the AED rightly recognised that shock delivery was not required.

**Table 2:** AED use: frequency and outcome

Total AED use	12	Successful defibrillations	Survived
**Defibrillations**	7	4	2
**No shock**	5	-	4

The mean time between turning on the units and application of the electrodes to the patient was 39 seconds (± 20 SD); 54 seconds elapsed (± 20 sec.) until the AED recommendation of "shock delivery" was displayed, and 96 seconds (± 20 sec.) elapsed until shock administration. Generally, proper function of the AEDs could be demonstrated in all cases. The unit protocols showed the proper technical procedure during use and the units gave automated speech announcements conforming to the guidelines.

In the course of the project, two lives may have been saved due to AED use leading to defibrillation. The estimated overall costs of the project are 651,380 € (see Table 3); however, the small numbers and study design do not allow proper estimation of the costs per saved life or quality-adjusted life years (QALYs).

**Table 3:** Approximate estimation of costs: 5-year AED project

**Purchase**	2000 € × 155 AEDs	310.000 €
**Maintenance**	100 € × 155 AEDs	15.500 €
**Training**	20 € × 6.924 individuals	125.880 €
**Hotline, surveillance, evaluation (labour costs)**	40.000 € × 5 years	200.000 €
**Total**		**651.380 €**

## Discussion

The present study reports five-year results of the first city-wide early defibrillation project in a German city. Our observation of proper AED function in all cases is consistent with other reports that modern AEDs from different providers are reliable in clinical use and enable quick, valid rhythm analysis and shock delivery if required [17,18]. Artefacts in AED recordings previously reported by others were not observed here [19]. The times between turning on the unit and announcement of shock delivery (mean value, 54 sec) and shock administration itself (mean value, 96 sec) are within the time ranges reported by other authors [20].

Every delay decreases the chance of successful defibrillation, as shown in our study in which a delay of 4-6 minutes until the arrival of firemen led to unsuccessful resuscitation in two cases requiring defibrillation. This has been confirmed by other authors; in the British National Defibrillator Programme, the installation of AEDs for public access defibrillation was clearly superior to the first responder strategy with transported AEDs [21]. In a study conducted by Cappucci et al. in the Piazenza region of Italy, the time lapse until the arrival of first responders in the vicinity and EMS was 4.8 min (± 1.2 min) and 6.2 min (± 2.3 min), respectively [22]. Generally, it must be considered that only about 1/3 of all sudden cardiac/circulatory arrests occur in public; the vast majority of these events occur in domestic environments [23]. The total number of 12 patients in this project seems relatively small considering that the incidence of treated primary arrests ranges from 37-100/100,000 annually according to a review by Chugh et al. [24]. This was confirmed by the ANPAD programme in which very few patients were reached, despite a relatively high number of installed devices [18]. Public access defibrillation is a highly effective strategy for patients with sudden cardiac arrest (SCA) in public places where AEDs are installed as shown in the British National Defibrillator Programme [20]. In that study, hospital discharge after SCA was achieved in over one-quarter of patients after the use of permanently installed AEDs. Deployment of AEDs reduces the time of the Call-to-the-First-AED-Prompt [25]. In the Public-Access Defibrillation Trial, the use of AEDs in communities was associated with a near doubling of survival after out-of-hospital cardiac arrest. These reports reinforce the importance of strategically expanding community-based AED programmes [26]. With regard to the strategic planning of AED placement, the recommendations of the European Resuscitation Council (ERC) differ from those of the American Heart Association (AHA); the ERC recommends AED installation in places where a cardiac/circulatory arrest is to be expected within two years, whereas the AHA recommends installation in places where a cardiac/circulatory arrest is to be expected within five years [27,28]. Earlier studies have suggested airports, highly frequented shopping centres, major businesses, sports clubs and recreational facilities [29,30]. In the literature, risk evaluation with regard to the relationship between use (including cost) and probable benefit has been recommended for the strategic planning of AED distribution [31]. According to a Danish study, high coverage of 10.6% of the city area was necessary to cover 66.8% of all cardiac arrests, whereas coverage of 1.2% only led to 10.6% of SCAs being reached [32]. Therefore, the AHA recommendations seem to be superior to the ERC guidelines.

Compared with other cities, e.g., Warsaw, the ratio of AEDs in Bochum is relatively high (one AED per 14,706 people in Warsaw vs. one per 2,451 citizens in Bochum) [33].

In Bochum, however, the current allocation of AEDs is still not comprehensive. Important sites for AED placement might have been omitted, and this could explain the relatively small number of 12 AED administrations during the project. The estimated costs of the project of more than 650,000 € for two saved lives within a five-year period is consistent with other investigations, showing that unguided placement is expensive and requires approximately threefold greater cost than strategically planned placement [34]. Training and equipping lay volunteers to use defibrillators, however, seems to be cost-effective compared to CPR training alone with regard to QALYs [28].

In the literature, it has been reported that the use of AEDs involves a high readiness to perform resuscitation [35-37]. According to the present data, resuscitation measures were performed by the AED user onsite for at least six of the eight patients who presented with pulseless cardiac/circulatory arrest. Clearly, the automated speech announcements of the unit were followed adequately with respect to the performance of cardiopulmonary resuscitation. This experience has been confirmed by observations made by other authors [38,35].

The present study has several limitations. At the beginning, due to its nature, the Bochum project was not based on a prospective study design with dedicated planning and a clear strategic determination of the number of AEDs, AED locations and the groups of persons involved and to be trained. It was designed as an initiative supported by different institutions with the potential for dynamic growth. Therefore, this study offers observational data from experiences during the implementation of a dynamic early defibrillation system project in a German city.

Due to the emergency medical and administrative structure in Germany, the EMS control room was informed about only a fraction of sudden deaths. Thus, valid data were not available regarding the frequency of sudden deaths, and representative data could not be collected in the scope of this study with regard to the time span between the occurrence of a cardiac/circulatory arrest and shock delivery. Although the time of alerting the EMS could be tracked, it was not possible to definitively conclude the timeline of the actual emergency events. In addition, the EMS trips reflected only a fraction of the total events. Furthermore, inaccurate data with regard to time were determined based on the AEDs used. For future analyses, the use of radio-controlled clocks in the AEDs should be considered for more accurate time analyses.

It must be mentioned that the training consisted mainly of one-time sessions, and follow-up training was only offered to some extent. There were no evaluations, so no conclusions can be drawn about the actual skill level of the involved individuals. In the future, follow-up training and training for an additional 500 persons are planned.

Despite these limitations, important conclusions can be drawn from this study for future projects. The combination of the "first responder" and "public access defibrillation programme" concepts appears to be reasonable. According to our experience, the training of a large number of first responders seems to be feasible within the given EMS structure and in other urban German areas. The coordination and high-quality management of such city-wide initiatives by a project office such as that affiliated with the EMS seems to be reasonable. The project office in this study performed 470 consultations via the hotline number during the observation period; therefore, the installation of a hotline seems to have been appropriate.

The relatively low number of 12 cardiac arrests within a period of 5 five years could be partly explained by the lack of comprehensive placement of AEDs in high-risk areas. The identification and equipping of high-public-access places according to the AHA recommendations (see above) is essential and will be realised with 35 additional AEDs within the next two years.

Since April 2009, information about the AED locations has been available from a central computer, and it has been possible to immediately alert first responders near the emergency site. Future analysis of the provided data and additional equipment for first responders (especially in the scope of major events) is expected to enable further improvements of this dynamic, continuing learning system.

It would be desirable to conduct large-scale prospective studies on city-wide early defibrillation projects in large cities to further improve the outcomes and cost-effectiveness of early defibrillation programmes.

## Competing interests

The authors declare they have no competing interests.
